# Hyperactive mTOR signals in the proopiomelanocortin-expressing hippocampal neurons cause age-dependent epilepsy and premature death in mice

**DOI:** 10.1038/srep22991

**Published:** 2016-03-10

**Authors:** Yuki Matsushita, Yasunari Sakai, Mitsunori Shimmura, Hiroshi Shigeto, Miki Nishio, Satoshi Akamine, Masafumi Sanefuji, Yoshito Ishizaki, Hiroyuki Torisu, Yusaku Nakabeppu, Akira Suzuki, Hidetoshi Takada, Toshiro Hara

**Affiliations:** 1 Department of Pediatrics, Graduate School of Medical Sciences, Kyushu University, Fukuoka 812-8582, Japan; 2 Department of Neurology, Graduate School of Medical Sciences, Kyushu University, Fukuoka 812-8582, Japan; 3 Division of Cancer Genetics, Medical Institute of Bioregulation, Kyushu University, Fukuoka 812-8582, Japan; 4 Division of Neurofunctional Genomics, Medical Institute of Bioregulation, Kyushu University, Fukuoka 812-8582, Japan

## Abstract

Epilepsy is a frequent comorbidity in patients with focal cortical dysplasia (FCD). Recent studies utilizing massive sequencing data identified subsets of genes that are associated with epilepsy and FCD. AKT and mTOR-related signals have been recently implicated in the pathogenic processes of epilepsy and FCD. To clarify the functional roles of the AKT-mTOR pathway in the hippocampal neurons, we generated conditional knockout mice harboring the deletion of *Pten* (*Pten*-cKO) in Proopiomelanocortin-expressing neurons. The *Pten*-cKO mice developed normally until 8 weeks of age, then presented generalized seizures at 8–10 weeks of age. Video-monitored electroencephalograms detected paroxysmal discharges emerging from the cerebral cortex and hippocampus. These mice showed progressive hypertrophy of the dentate gyrus (DG) with increased expressions of excitatory synaptic markers (Psd95, Shank3 and Homer). In contrast, the expression of inhibitory neurons (Gad67) was decreased at 6–8 weeks of age. Immunofluorescence studies revealed the abnormal sprouting of mossy fibers in the DG of the *Pten*-cKO mice prior to the onset of seizures. The treatment of these mice with an mTOR inhibitor rapamycin successfully prevented the development of seizures and reversed these molecular phenotypes. These data indicate that the mTOR pathway regulates hippocampal excitability in the postnatal brain.

Epilepsy, which affects 0.8–1% of the world’s general population, is a leading cause of neurological problems in childhood^1^,^2^,^3^. The prevalence rate is even higher individuals with autism spectrum disorder (ASD)^4^,^5^ and various brain malformations^6^,^7^. The high prevalence of epilepsy in children with these disorders has led neurologists to investigate commonalities in their genetic backgrounds. However, searching for such genetic factors is challenging because diverse sets of genetic variations are known to be associated with the onset of both epilepsy and ASD^8^,^9^. On the other hand, syndromic phenotypes of Mendelian disorders have provided clues to elucidate their common pathogenic mechanisms.

The mammalian target of rapamycin (mTOR) constitutes an important cascade which regulates cell growth, differentiation and metabolism. Accordingly, mTOR signaling disorders are implicated in various diseases, including cancer, epilepsy and ASD^10^. Somatic mutations in the *PIK3CA*, *AKT3* and *mTOR* genes were recently identified to cause hemimegalencephaly^11^ and focal cortical dysplasia type II^12^,^13^. Thus these studies demonstrated that hyperactive mTOR signaling was involved in neuronal hypertrophy, aberrant circuit formation and epileptogenesis.

*Phosphatase tensin homolog* (*Pten*) encodes a lipid phosphatase that counteracts PI3K and AKT as an upstream regulator of mTOR. PTEN not only prevents overgrowth and tumorigenesis in proliferating cells, but also protects neurons from hyper-excitability and epileptogenesis^14^,^15^. Mouse models have been created, recapitulating progressive hypertrophy of the brain, epilepsy and autistic behaviors, which are the core symptoms of patients with Cowden syndrome and related human diseases^16^,^17^.

Proopiomeranocortin (Pomc), the precursor peptide of adrenocorticotropic hormone (ACTH), is expressed in the particular subsets of neurons in the hippocampal dentate gyrus (DG), hypothalamic arcuate nucleus and amygdala^18^. In the hippocampal DG, for example, Pomc is only transiently expressed in newly born progenitors of granule cells and immature neurons^19^,^20^. It is also noteworthy that deficits in ACTH (POMC) production are implicated as an underlying cause of infantile-onset epileptic encephalopathy^21^, a heterogeneous group of neuro-developmental disorders in childhood characterized by intractable epilepsy and unfavorable developmental outcomes^22^. Thus, we hypothesized that certain molecular pathways might regulate the proper expression of POMC in the hippocampus, thereby preventing the hippocampal neurons from hyper-excitability at an early stage of the postnatal brain.

In the present study, we explored the functional impact of hyperactive mTOR signaling on hippocampal neurons in the postnatal period. Using the *Cre-loxP* system, we deleted the murine *Pten* gene from hippocampal neurons expressing Pomc. All of these mice developed spontaneous seizures at postnatal week 8–10. Our data provide new lines of evidence that the deregulation of the mTOR pathway in the hippocampal Pomc-expressing neurons contributes to the onset of age-dependent epilepsy.

## Results

### Increased PI3K-Akt signaling in the *Pten*-cKO hippocampal dentate gyrus

**Figure 1:**
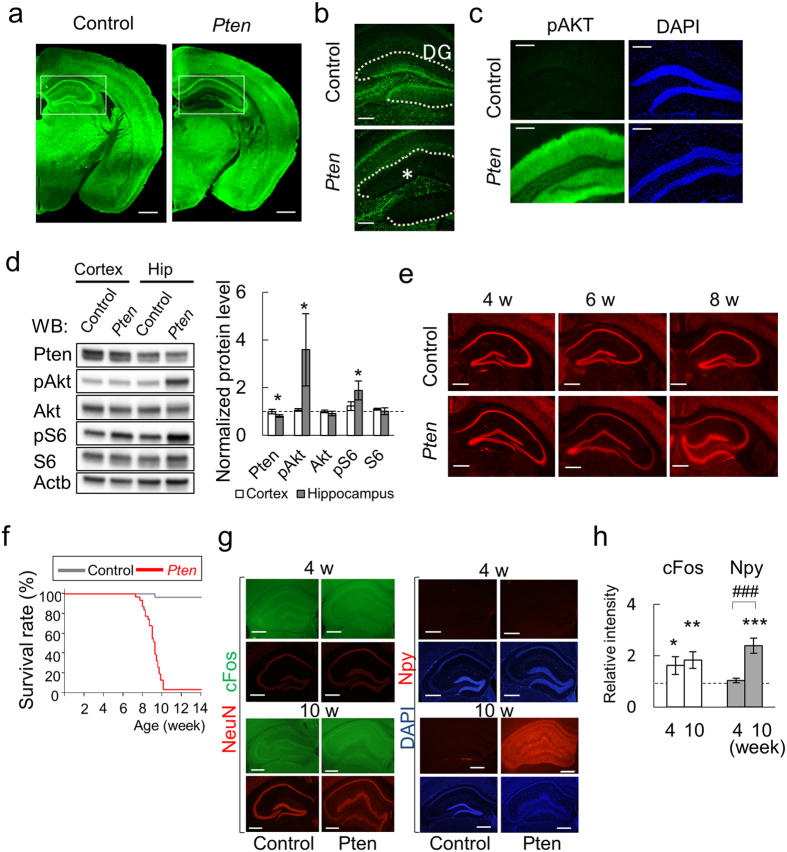
The characteristics and morphological findings of *Pten*-cKO mice. (**a**) Immunofluorescence studies on Pten expression (green). The left two panels show the coronary brain sections from control (left) and *Pten*-cKO (right, *Pten*) mice at 4 weeks of age. The squares denote the areas of the hippocampus DG. Scale bar, 1 mm. (**b**) Confocal images with a higher magnification of the DG. Asterisk indicates the loss of Pten signals (green) in the granule cell layer of DG (dashed lines). Scale bar, 200 μm. (**c**) Hyperactive AKT signaling in the DG of *Pten*-cKO mice. The immunofluorescence signals of the phosphorylated S473 of AKT (pAKT, green) and DAPI (blue) are shown. Scale bar, 200 μm. (**d**) Western blotting of the cerebral cortex and hippocampal extracts from control and *Pten*-cKO mice at 8 weeks of age. The bar plots for the relative expression levels of the indicated proteins are shown in the right panel. Note that increased pAKT and pS6 signals are seen in the hippocampus, but not in the cerebral cortex of *Pten*-cKO. *P < 0.05 (n = 4–6, Wilcoxon rank sum test). (**e**) Progressive hypertrophy of the DG in *Pten*-cKO mice. The morphological changes of the DG hippocampal sections at the ages of 4, 6 and 8 weeks. See Supplementary Fig. S1 for the whole brain images. Scale bar, 500 μm. (**f**) Survival rates (%) of control and *Pten*-cKO mice. (**g**) cFos (green) and NeuN (red) expression (left panels). Npy (red) and DAPI (blue) expression (right panels), in the DG at 4 and 10 weeks of age. Scale bars, 500 μm. (**h**) The quantitated results of (**f,g**). The relative signal intensity (*Pten*-cKO/control) of cFos and Npy at 4 and 10 weeks are plotted. Asterisks indicate significantly higher levels of cFos and Npy expressions in the DG of Pten-cKO mice in comparison to the control mice. P < 0.05, **P < 0.01, ^###^ and ***P < 0.001 (n = 4–16, Wilcoxon rank sum test).

We first investigated the regional expression of Pten in the *Pten*-cKO mice and their littermate controls. Immunofluorescence studies showed that Pten signals were not present in the hippocampus DG or the hypothalamus arcuate nucleus (Arc) of the *Pten*-cKO mice: whereas Pten was abundantly expressed in the control mice of the same age (Fig. 1a,b, Supplementary Fig. S1). Phosphorylated Akt (pAkt) and S6 (pS6) signals were consistently increased in these regions in the *Pten*-cKO mice, but not in the control mice (Fig. 1c). These data clearly demonstrated that the PI3K-Akt pathway hyperactivity was limited to the area of the hippocampal DG and the hypothalamus. Furthermore, quantitative Western blotting analyses showed that the pAkt and pS6 signals in the hippocampus of *Pten*-cKO mice were stronger than those in the control mice (Fig. 1d). Such differences were not observed in the cerebral cortex extracts from the *Pten*-cKO and control mice.

### *Pten*-cKO mice exhibit age-dependent seizures and premature death

The vital conditions and somatic growth of many of the brain-specific *Pten* knockout mice are reported to be severely affected from the early postnatal period^17^,^23^. We therefore continued to observe their gain of weight and general activity. Our *Pten*-cKO mice grew normally and their body weight gain was not significantly different to that of control mice (Supplementary Fig. S1). Although the appearance of the brain in *Pten*-cKO mice did not differ from that of control mice, the whole brain was significantly heavier (n = 18 and 15, respectively. P = <0.0001, Wilcoxon rank sum test, Supplementary Fig. S1). We serially examined the brain sections of the control and *Pten*-cKO mice at 4, 6 and 8 weeks of age. We noticed that the hippocampus of the *Pten*-cKO mice gradually became enlarged and distorted in an age-dependent manner (Fig. 1e, Supplementary Fig. S1).

Notably, all (n = 31) of the *Pten*-cKO mice became hypersensitive and began to show a rigid posture with startle responses to the routine handling during cage exchange from 8 weeks of age. Ninety-seven percent (30 of 31) of these mice died by the tenth week of age (Fig. 1f). Since epilepsy is commonly observed in ASD patients and animal models^4^,^24^, we wondered whether the sudden onset of this phenotype might be associated with abnormal neuron excitability. Indeed, in *Pten*-cKO mice, the hippocampal DG expressed significantly higher levels of cFos, a neuronal activity marker, than in control mice of the same age (P = 0.0028, Fig. 1g,h). Neuropeptide Y (Npy) was over-expressed at this age in the DG of the *Pten*-cKO mice in comparison to the control mice (P < 0.0001, Fig. 1g,h). We also verified that there were no sex differences in the age of onset or morbidity between the control and *Pten*-cKO mice. Double-heterozygous mice (*Pomc-Cre*^*Tg*/+^;*Pten*^*flox*/+^) did not show the seizure phenotype, which excluded the deleterious effects of *Pomc-Cre* transgene (n = 13). These data strongly suggested that the congenital loss of *Pten* in the DG might cause a highly penetrant phenotype of epilepsy in mice at the eighth to tenth week of age.

### *Pten*-cKO mice present spontaneous seizures at eight to ten weeks of age

**Figure 2:**
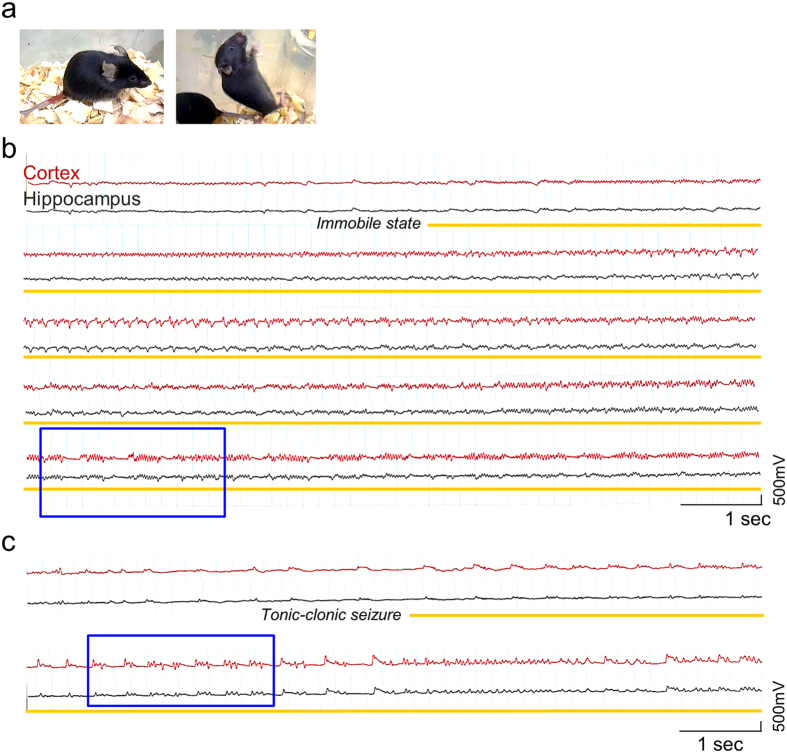
Video-monitored EEG recordings of seizures in *Pten*-cKO mice. (**a**) *Pten*-cKO mice in static (left) and generalized tonic-clonic seizures (right). (**b,c**) Representative EEG traces associated with seizures in *Pten*-cKO mice. Cortical (red) and hippocampal (black) electrodes detected the transition of resting background activity evolving into the paroxysmal phase (yellow lines). Blue squares denote intervals, in which high-amplitude, rhythmic poly-spike (**b**) or periodic bursts (**c**) are seen.

When closely inspecting the behaviors of *Pten*-cKO mice under video monitoring, we found that recurrent seizures began to occur at 8–10 weeks of age. These seizures were classified into two types: an immobile state with unresponsiveness (Fig. 2a left, Supplementary Video S1) and generalized tonic-clonic convulsions accompanying intermittent falls and jumping (Fig. 2a right, Supplementary Video S2). We thus conducted simultaneous video-electroencephalography (EEG) recordings of *Pten*-cKO mice at 8–10 weeks of age after the insertion of stereotactic electrode. Two channels of electrodes were used to monitor the electrical activity of the hippocampus and cerebral cortex (Supplementary Fig. S2). Epileptiform discharges rarely appeared in the interictal phase (Fig. 2b), whereas ictal patterns of rhythmic sharp waves, poly-spikes (Fig. 2b, blue square), and periodic suppression and burst patterns of epileptiform discharges (Fig. 2c, blue square) emerged concurrently with the seizures (Racine’s score at IV/V).

No preceding epileptiform discharges were evoked in the hippocampus prior to the activation of the cerebral cortex. Thus it was suggested that epileptogenic circuits between the two regions already existed when the *Pten*-cKO mice exhibited seizures. To biochemically establish whether the hippocampus was the epileptogenic origin in our *Pten*-cKO mice, we examined whether hippocampal *cFos* expression might precede the phenotypic onset of seizures. The time course of *cFos* expression showed that *cFos* mRNA was robustly induced in the hippocampus of the *Pten*-cKO mice at 8 weeks of age: it was not induced in the cerebral cortex (Supplementary Fig. S3). Increased *cFos* expression in the cerebral cortex was observed at 9–10 weeks of age, during the period of the phenotypic onset of seizures (Supplementary Fig. S3). Such transcriptional activation was never observed in the hippocampus or the cerebral cortex of the control mice.

### The excitatory-inhibitory synaptic imbalance in the hippocampus of *Pten*-cKO mice

**Figure 3:**
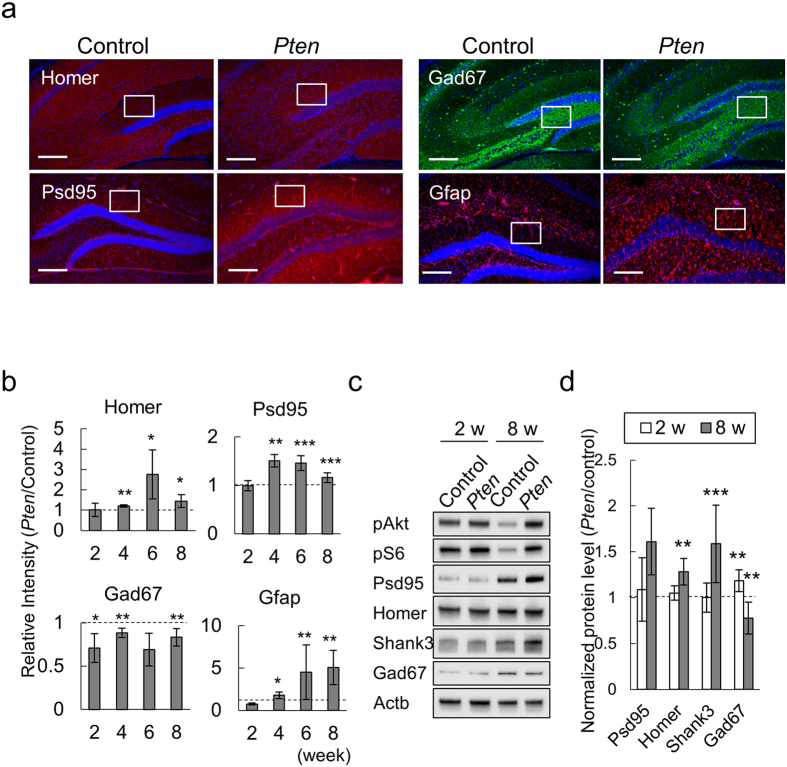
Excitatory-inhibitory imbalance in the hippocampus of *Pten*-cKO mice. (**a**) The expression of excitatory synapse (Homer, Psd95), inhibitory neuron (Gad67) and glial markers (Gfap) in the hippocampus of control and *Pten*-cKO mice at 6 weeks of age. Immuno-labeled proteins are annotated. Squares indicate the ROIs for the quantitative analyses. Scale bar, 200μm. (**b**) The quantitated results of the immunofluorescence studies. The relative fluorescence intensity (*Pten*-cKO/Control) of Homer, Psd95, Gad67 and Gfap are plotted for 3 or more independent pairs of *Pten*-cKO and control littermates at each time point. Horizontal dashed lines indicate the reference values of the control mice. (**c**) Representative images of western blots for the hippocampal extracts. Three or more pairs of control and *Pten*-cKO mice at 2 and 8 weeks of age were used for this study. (**d**) The quantitated Western blotting data. Bar plots for the relative expression (*Pten*-cKO/Control) of the indicated proteins. The plotted values show the means ± SD (n ≥ 3). In panels (**b,d**), *P < 0.05, **P < 0.01, ***P < 0.001.

To gain insight into the molecular bases for the age-dependent seizures that occurred in our *Pten*-cKO mice, we tested whether excitatory-inhibitory (EI) imbalances might contribute to their pathogenic process. Serial immunofluorescence studies revealed that the DG of *Pten*-cKO expressed significantly higher levels of excitatory synapse markers, Homer and Psd95, in parallel with an astroglial marker, Glial fibrillary acidic protein (Gfap), than the control mice by 6 weeks of age (P = 0.021, 0.0004, 0.0011, respectively; Fig. 3a,b). On the other hand, Gad67, a GABAergic interneuron-specific marker was expressed at a lower level in the hilus region of the DG of the *Pten*-cKO than in control mice (P = 0.2817, Fig. 3a,b). The time course of these data supported that the differential expressions of excitatory and inhibitory neurons in the DG of the *Pten*-cKO mice were already evident prior to the onset of seizures (Fig. 3b), suggesting that the excitatory and inhibitory imbalance might be a prerequisite for the epileptogenic condition of the *Pten*-cKO mice. Western blotting also supported the expression levels of Homer and Shank3 were significantly higher in the hippocampus of the *Pten*-cKO mice than in the control mice at 8 weeks of age, but not at 2 weeks of age (Fig. 3c,d).

### Age-dependent hypertrophy of the dentate gyrus neurons in *Pten*-cKO mice

**Figure 4:**
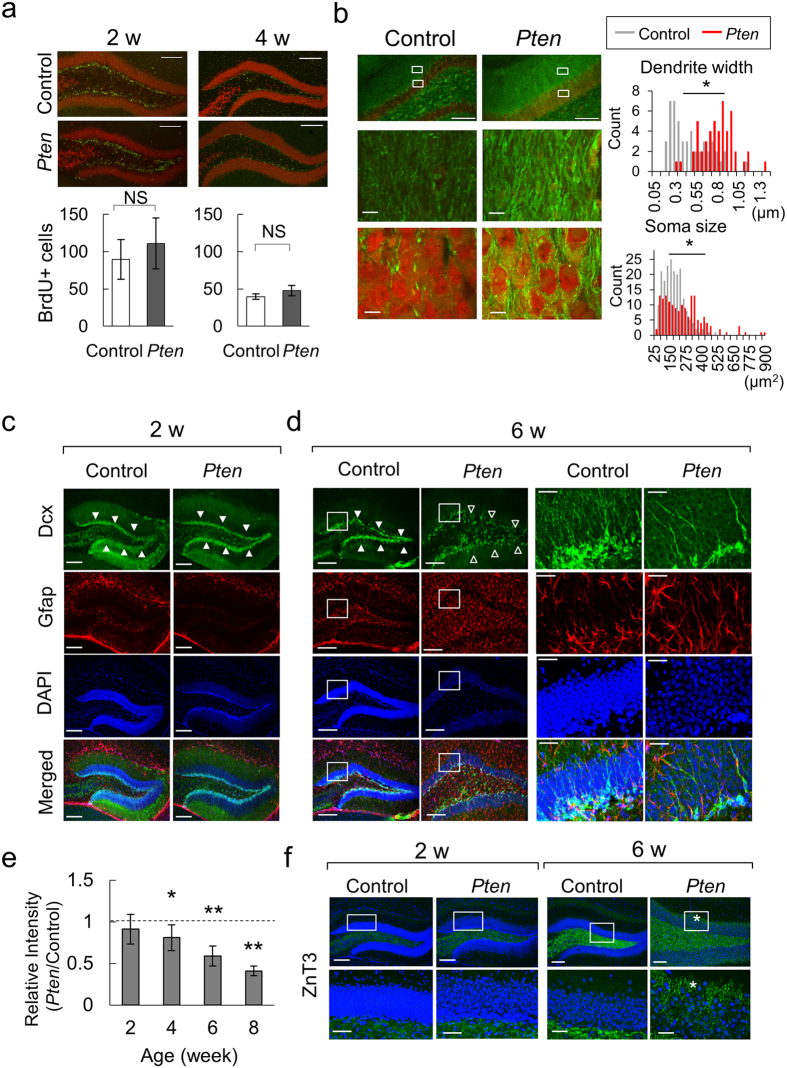
Hypertrophy of the DG and dysmorphic neurons in *Pten*-cKO mice. (**a**) BrdU (green) and NeuN (red) signals in the DG of the control and *Pten*-cKO mice at 2 and 4 weeks of age. The number of BrdU-positive cells in the subgranular zone are plotted (means ± SD values, n = 3). NS, not significant. Scale bars (**a–d**) denote 200 μm unless otherwise stated. (**b**) Hypertrophy of the DG neurons in the *Pten*-cKO mice. Left images show Map2 (green) and NeuN (red) signals. Histograms represent the counts for indicated widths of dendritic shafts (right upper, 50 for control and 50 for *Pten*-cKO) and the sizes of soma (right lower, 200 control and 160 *Pten*-cKO) from 3 pairs of littermates are shown. *P < 0.0001. Scale bars (lower panels), 10 μm. (**c,d**) Aberrant differentiation of neuronal progenitors in the DG of *Pten*-cKO. Dcx (green), Gfap (red) and DAPI (blue) signals are shown. Filled arrowheads indicate the Dcx signals peaked at the subgranular zone. Lucent arrowheads point to the sparse patterns of the Dcx signals at 6 weeks of age (d, *Pten*-cKO). Higher magnification views are provided for the boxed (d, far-right two columns). Scale bars, 20 μm (**d**) higher magnification images). (**e**) The relative fluorescence intensity (*Pten*-cKO/control) of Dcx in the subgranular zone at 2, 4, 6 and 8 weeks of age (mean ± SD, n ≥ 3). (**f**) Znt3 (green) and DAPI (blue) signals in DG at 2 and 6 weeks of age. Asterisks indicate the presence of abnormal sprouting of mossy fibers. The lower panels show higher magnification views of the boxed areas. Scale bars, 50 μm (lower panels).

We investigated whether the progressive EI imbalance in the DG of *Pten*-cKO mice might be chronologically associated with its morphology after birth. To distinguish hypertrophy of the differentiated neurons from overly proliferating stem cells, we performed bromodeoxyuridine (BrdU) labeling of the neural stem cells in the DG, and compared the numbers in the *Pten*-cKO and control mice at 2 and 4 weeks of age. Contrary to our prediction, there was no difference in the number of proliferating neural stem cells in the *Pten*-cKO and control mice (Fig. 4a). The microtubule-associated protein 2 (Map2)-labeled dendritic shafts of neurons in the molecular layer in the *Pten*-cKO mice were observed to be significantly thicker than in control mice (Fig. 4b, upper and Supplementary Fig. S4). NeuN-positive neurons in the granular cell layer (GCL) showed a significantly larger soma (Fig. 4b, lower). We thus considered that the hypertrophy of the *Pten*-cKO DG was mainly the result of aberrant neuronal differentiation, and had not been caused by an excess of self-proliferating neural stem cells.

To dissect the differentiation process of progenitor cells, we consecutively analyzed the cell populations by immuno-labeling for doublecortin (Dcx) and Gfap in *Pten*-cKO and control mice at 2, 4, 6 and 8 weeks. At two weeks of age, the numbers of Gfap and Dcx-positive cells in the *Pten*-cKO mice did not differ from the control mice (Fig. 4c). As previously mentioned, the numbers of Gfap-positive cells in the DG of the *Pten*-cKO mice were increased at 6–8 weeks of age (Figs 3b,4d and Supplementary Fig. S5). On the other hand, the number of Dcx-positive progenitor cells in the *Pten*-cKO mice decreased to 65% of the number in the control mice at 6 weeks of age (Fig. 4c–e and Supplementary Fig. S5). Another prominent feature was the Dcx-positive progenitors were dispersed in the DG of *Pten*-cKO mice at 6–8 weeks of age. These data indicated that loss of *Pten* in the hippocampal DG disturbed neuronal differentiation.

The improper differentiation of neuronal progenitor cells prompted us to investigate whether *Pten*-cKO mice might develop abnormal neural circuits after birth. To confirm this, we analyzed the sprouting patterns of mossy fiber, the outward axon from the granule cells of the DG, projecting to the CA3 region^25^. An immunofluorescence study with an axonal marker, Znt3, verified that the mossy fibers in the hilus region in both control and *Pten*-cKO mice were normal at 2 weeks of age (Fig. 4f, left). However, the Znt3 signals of the *Pten*-cKO mice penetrated the granule cell layer and the fibers extended to reach the molecular layer at 6 weeks of age (Fig. 4f, right). Thus, our microscopic data confirmed that the regional *Pten* deletion in the Pomc-expressing neurons caused impaired neuronal differentiation in the DG, which was accompanied by progressive hypertrophy and excessive excitatory synapse components after birth. Moreover, the aberrant morphology of differentiating neurons in the DG coincided with abnormal patterns of dendritic polarity and mossy fiber sprouting from granule cells. These data indicated that the hippocampus of the *Pten*-cKO mice already formed epileptogenic circuits prior to the onset of seizures.

### Neuroendocrine dysfunctions in the hippocampus of *Pten*-cKO mice

We further sought the mechanisms that disturbed the homeostasis of the EI balance in our *Pten*-cKO mice. Adrenocorticotropic hormone (ACTH), a cleavage product of POMC, is the most effective therapeutic agent for treating epileptic encephalopathy in childhood^22^. We therefore hypothesized that our *Pten*-cKO mouse might be a relevant model of the deregulation of the corticotropin releasing hormone (CRH) - ACTH axis.

We analyzed the expression of *Crh* and *Pomc* mRNAs in the hippocampus of *Pten*-cKO mice at different time points before and after the onset of seizures, and compared them with control mice. We found that the hippocampus of the *Pten*-cKO mice already expressed significantly higher levels of *Crh* mRNA at 8 weeks of age (8 vs. 4 weeks of *Pten*-cKO, P = 0.0034, Supplementary Fig. S6). The *Crh* expression rose even higher after the onset of seizures (9–10 vs. 4 weeks, P = 0.0058, Supplementary Fig. S6). In contrast, *Pomc* expression declined in the hippocampus of the *Pten*-cKO mice as they grew older, but remained unchanged in the hippocampus of control mice. Consequently, the *Pten*-cKO mice expressed significantly lower levels of *Pomc* mRNA after the onset of seizures than they did before the onset of seizures (9–10 vs. 4 weeks, P = 0.0137, Supplementary Fig. S6). There was also a significant difference in the Pomc expression of the *Pten*-cKO and control mice at 9–10 weeks of age. The loss of *Pten* in the hippocampus did not affect *Crh* expression in the cerebral cortex or the hypothalamus (Supplementary Fig. S6), which excluded the possibility that increased *Crh* in the hippocampus was only a secondary effect. These findings indicated that the loss of *Pten* in the Pomc-positive neurons disturbed the regulation of *Crh* and *Pomc* expression in the developing hippocampus, rather than in the hypothalamus or cerebral cortex. It was also consistent with a feedback-loop model of elevated CRH due to the decreased synthesis of ACTH (POMC/MSH) in the pathogenic condition of human epileptic encephalopathy^21^.

Nonetheless, genetic expression and subsequent releases of neuropeptides from cells are known to fluctuate with circadian rhythm and other chronological factors^26^. It was therefore a possibility that the subtle differences in the expressions of neuropeptides be a consequence of variable conditions in the analysis. We therefore analyzed the co-expression profiles of *Crh* with *Crh receptor 1* (*Crhr1*) at 8–10 weeks. Intriguingly, the *Crh-Crhr1* correlation plots showed distinct patterns of co-expression in the 3 regions that were tested in the control and *Pte*n-cKO mice (Supplementary Fig. S6). These data convinced us that both an excess of Crh and a shortage of Pomc became prominent in the hippocampus of the *Pten*-cKO mice at the age of onset (8–10 weeks), but not before (4–6 weeks of age).

To assess the functional relationship between mTOR hyperactivity and the deregulated Crh-ACTH axis in our *Pten*-cKO mice, we attempted to control the seizures by treating them with serial intraperitoneal injections of ACTH (ACTH 1–24, Sigma A0298, 0.0125 mg/kg/day for seven consecutive days at 8 weeks of age). However, the ACTH therapy did not improve their seizures or mortality rate (our unpublished results). It was therefore unlikely that neuroendocrine deficits played a major role in the onset of epileptic seizures in our *Pten*-cKO mice.

### Rapamycin restores the epileptogenic phenotypes of *Pten*-cKO mice

**Figure 5:**
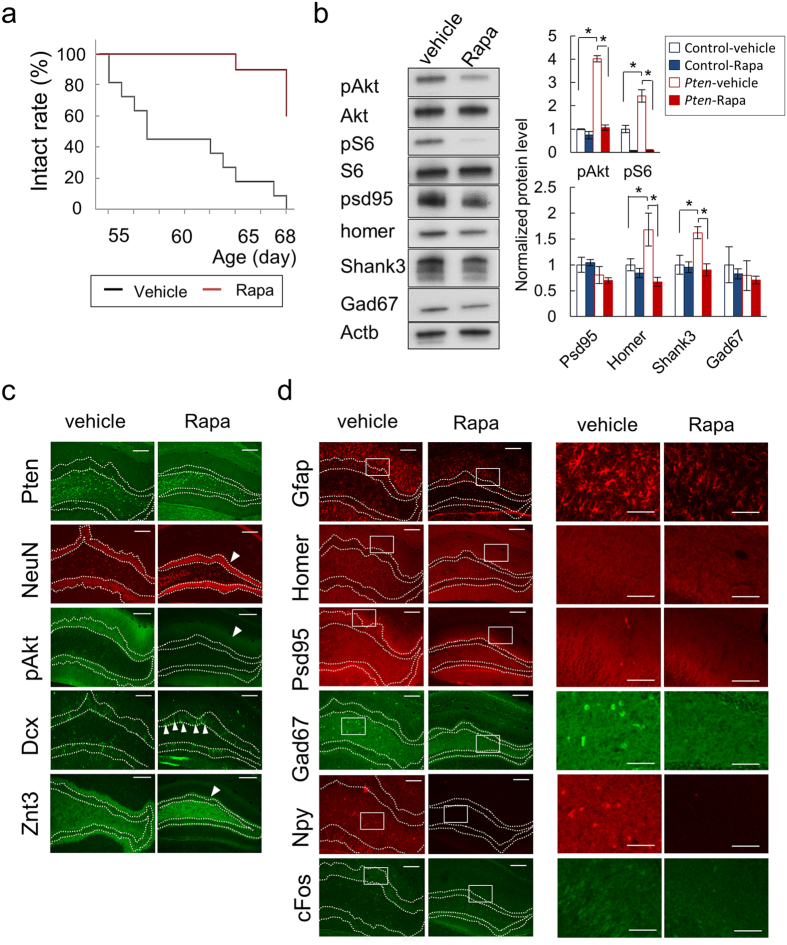
The postnatal treatment of *Pten*-cKO mice with rapamycin extends their intact survival and normalizes their molecular phenotype. (**a**) Intact survivals (%) of *Pten*-cKO mice with vehicle or rapamycin (Rapa) treatment. (**b**) Western blotting for the hippocampal extract from vehicle- or rapamycin-treated *Pten*-cKO mice. The bar plots show the quantitative results (mean ± SD) of Western blotting from 3 pairs of mice with or without rapamycin treatment. The expression level of the control mice after the vehicle treatment was set as 1 (reference). *P < 0.05. (**c,d**) Immunofluorescence images of the DG from vehicle or rapamycin-treated *Pten*-cKO mice at 9 weeks of age. The arrowheads indicate the presence of neurons in which the expression of annotated proteins recovered after rapamycin treatment (**c**). Higher magnification images of the boxed areas are provided in the two columns on the right (**d**). Dashed lines denote the boundaries of the granule cell layers of the DG (**c,d**). Scale bar, 200 μm and 100 μm ((**d**) higher magnification).

Lastly, we determined whether the epilepsy of the *Pten*-cKO mice could be mitigated by controlling the hyperactive mTOR signaling with its inhibitor rapamycin. *Pten*-cKO mice were treated with vehicle or rapamycin from 6 to 9 weeks of age, and their brains were subjected to Western blotting and immunofluorescence studies. We found that the rapamycin-treated brains weighed significantly less than vehicle-treated brains (P = 0.0015, Supplementary Fig. S7). Rapamycin successfully prevented *Pten*-cKO mice from developing seizures, and the seizure-free survivals of the vehicle and rapamycin-treated *Pten*-cKO mice were 0% (0 of 11) and 82% (9 of 11), respectively (P < 0.0001, Fig. 5a). Western blotting showed that rapamycin treatment suppressed the increased pAKT and pS6 signals in the hippocampal extracts from the *Pten*-cKO mice to the level of the control mice (Fig. 5b, Supplementary Fig. S7). More intriguingly, the expression of Homer and Shank3 was normalized to the control level.

Microscopic data also supported that the treatment with rapamycin suppressed the hypertrophy of the DG as well as the excessive pAKT signaling in *Pten*-cKO mice (Fig. 5c: NeuN and pAKT). We verified that the loss of Pten expression in this region did not differ in the treatment and non-treatment groups (Fig. 5c: Pten). Rapamycin treatment led to a recovery of Dcx expression with peak signal intensity at the subgranular zone of the DG (Fig. 5c: Dcx). Concordant with these data, rapamycin blocked the abnormal protrusion of mossy fibers into the granule cell layers (Fig. 5c: Znt3). Regarding the EI imbalance in the *Pten*-cKO mice, the abnormal distribution of Gfap, Homer and Psd95 in this area was corrected (Fig. 5d: Gfap, Homer and Psd95). While the expression of interneuron marker, Gad67, was significantly reduced, the reactive expression of Npy was nearly completely absent from the DG after rapamycin treatment (Fig. 5d: Gad67 and Npy). Lastly, we verified that rapamycin suppressed the expression of cFos, indicating that the treatment successfully controlled the excitability of the DG neurons in the *Pten*-cKO mice.

## Discussion

Our *Pten*-cKO mice developed spontaneous seizures at a relatively mature stage (8–10 weeks) in comparison to brain-wide or DG neuron-specific knockout mice^16^,^17^,^24^,^27^. Among the various types of brain-specific *Pten* knockout mice, our *Pten*-cKO mice resembled those of previous reports^28^,^29^. Our experimental data recapitulated their major findings and showed some novel findings: 1) the inborn deletion of *Pten* in the hippocampal DG was sufficient to cause severe epilepsy in adulthood; 2) the hyperactive mTOR signaling pathway disrupted Crh-ACTH homeostasis in the hippocampus; and 3) postnatal treatment with rapamycin not only reversed the seizure phenotype, but also corrected the molecular phenotypes of the excessive production of ASD-associated proteins, such as Shank3 and Homer.

Ljungberg *et al.* demonstrated the value of neuron subset-specific *Pten* knockout mice as an animal model for focal cortical dysplasia (FCD) and the antiepileptic effects of rapamycin^28^,^29^. Since our *Pten*-cKO mice harbored genetic mosaicism, which resulted in the chimeric expression of *Pten* in their brain, they can be also regarded as a disease model for congenital disorders with hyperactive AKT-mTOR conditions due to somatic mutations. Patients with FCD, a brain malformation, suffer from intractable epilepsy in childhood and early adulthood^30^. The diagnostic categories of FCD are based on distinct features in neuroimaging studies and the histopathological findings of surgically resected tissues^31^. Among these categories, FCD type II is characterized by the presence of cytomegalic dysmorphic neurons and balloon cells^30^,^32^.

Both cytomegalic dysmorphic neurons and balloon cells have been considered analogous to giant cells in the tuberous sclerosis complex (TSC), which is another example of a Mendelian disorder which causes epilepsy and developmental problems in childhood. Indeed, recent studies have shown balloon cells to be characteristically hyperactive in the mTOR pathway^33^. In line with these biological notions, recent evidence has shown that balloon cells carry somatic mutations in genes encoding components of the AKT-mTOR pathway^12^,^13^. Moreover, these cells have been suspected to function as generators of paroxysmal activity in epileptogenic origin^34^. Thus, it was reasonable that our *Pten*-cKO mice exhibited age-dependent seizures associated with regionally hyperactive AKT-mTOR signals in the hippocampus, wherein the *Pten*-deficient hypertrophic neurons were likely epileptogenic sources.

It remains controversial, however, whether our *Pten*-cKO mice can be considered as a new model for FCD. Alternatively, they may be a better fit for a phenotype of temporal lobe epilepsy (TLE) in adulthood^35^. Although the majority of cases with TLE are known to show hippocampal atrophy or sclerosis, earlier studies using biopsy and autopsy samples demonstrated the hypertrophy of hippocampal DG neurons^36^. More notably, hypertrophy of the DG in TLE patients appeared to show sparsely scattered appearance of DCX-positive immature neurons^37^ and accelerated AKT-mTOR signaling^28^. Thus, excessive AKT-mTOR signaling might contribute to the neuronal hypertrophy of the DG neurons at an early stage of TLE, while they eventually follow the course of degeneration with additive effects of chronic excitotoxicity, secondary inflammations and other molecular mechanisms. To validate this hypothesis, it could be worth testing whether the hypertrophic neurons in our *Pten*-cKO mice will eventually exhibit sclerotic degeneration when they are successfully rescued to survive after the onset of seizures.

Aberration of the Crh-ACTH axis has been implicated in the onset of infantile spasms (IS), the most prevalent form of epileptic encephalopathy in infancy^21^. We thus initially hypothesized that loss of *Pten* in Pomc-expressing neurons could reproduce child-onset seizures that resemble the phenotypes of humans. However, our *Pten*-cKO mice did not show such early-onset seizures. These data raised two possibilities: that the Pomc-positive neurons of the hippocampus might be irrelevant to the pathogenic process of IS: or that the hippocampal neurons could be relevant, but insufficient to drive similar phenotypes in mice. We emphasize the value of the latter hypothesis because previous studies supported this concept. For example, a clinical study using single photon emission tomography revealed decreased regional cerebral blood flow in the hippocampus of IS patients^39^. In rodents, ACTH was shown to regulate the hippocampal expressions of *Crh* and *Crhbp*^40^. Furthermore, considering the various types of mouse models of IS that have been generated to date, their ages of onset and seizure patterns did not necessarily correlate to those of humans. These findings pointed to the fact that the creation of rodent models of IS remains challenging due to technical difficulties^41^.

The cleavage product of POMC, alpha-melanocoritin stimulating hormone (alpha-MSH), exerts anorectic functions through its binding to central melanocortin receptors (MCRs) that are expressed in the hypothalamic arcuate nucleus^42^. Different types of MCRs are also expressed in the central nervous systems of mammals. Among them, MC3R and MC4R have been reported to bind to both ACTH and MSH, and to modulate hippocampal synaptic plasticity^43^. Intriguingly, the functional loss of MC4R resulted in cognitive dysfunctions, suggesting the indispensable roles of MSHs and ACTH for cognitive development in childhood^44^. Moreover, early intervention with ACTH treatment has been shown to have benefits on the long-term prognosis of seizures as well as cognitive development in children affected by IS^45^.

In this study, we found that the loss of *Pten* not only caused a regional EI imbalance and impaired the differentiation of neuronal progenitors, but that it also disturbed the synthesis of *Pomc* itself in the hippocampus after birth. These data collectively suggested that the mTOR pathway in Pomc-expressing neurons might contribute to both cognitive development and epileptogenesis.

We found that the postnatal treatment of our *Pten*-cKO mice with rapamycin was effective for preventing the onset of their epilepsy. Recent studies have also shown that administration of mTOR inhibitors ameliorated epilepsy in FCD and tuberous sclerosis^46^,^47^. These data indicate that the homeostatic regulation of the mTOR signals is essential for the development of functional connectivity in the postnatal brain. However, it is unclear how the hyperactive mTOR pathway contributes to the formation of epileptogenic circuits in a certain period of childhood. Both cell-intrinsic and non-intrinsic models have been proposed as epileptogenic mechanisms in FCD^48^,^49^. In this study, we found that the expression levels of ASD-associated proteins, such as Shank3 and Homer, fluctuated according to the hyperactive or normally-regulated conditions of the mTOR signals. Thus, it can be hypothesized that hyperactive mTOR signals might lead neurons to become hyper-connective through the excessive biogenesis of excitatory synapses, as proposed in the pathogenic model for the ASD brain^50^,^51^. These findings appeared to be consistent with those of our previous studies, which predicted that the Shank-Homer protein complexes might regulate the activity of the mTOR pathway in the postsynaptic compartments^52^. *Pten* and *Shank* double mutants will clarify the functional roles of ASD-associated proteins in the development of epilepsy in adults.

Children with tuberous sclerosis complex are highly susceptible to IS, while they develop ASD later in childhood^53^. Thus, one could speculate that the pathogenic processes underlying epileptic encephalopathy may share common molecular pathways with ASD, but that such molecular pathways may contribute to the phenotypic onsets through distinct subsets of neurons or differential brain regions. Identifying downstream molecular pathways, such as the AKT-FOXG1 axis, will be the key to elucidating the reason why developmental delay and autism are common sequelae in patients with IS and other forms of epileptic encephalopathy^46^.

In conclusion, this study disclosed that the Pomc neuron-specific loss of *Pten* was sufficient to cause age-dependent seizures in mice. This study also supported that hyperactive mTOR signaling contributed to the development of epileptogenic circuits through the overproduction of excitatory synapse-associated proteins. Searching for events downstream of the mTOR pathways will provide further benefits in future translational research for patients with FCD and associated diseases.

## Materials and Methods

### Ethics statement

All experimental methods were carried out in accordance with the approved guidelines by Institutional Review Board and licensing Committee at Kyushu University (#23–53). All of the mouse experiments were performed according to guidelines and protocols which were approved by the Kyushu University Institutional Animal Care and Use Committee (#A-25–006).

### Animals

Mice were housed in a specific pathogen-free environment and maintained in a C57BL/6 J background. The animals had *ad libitum* access to food and water at all times, the temperature was maintained at 25 °C with a 12-h light-dark cycle. Sex-matched littermates were used for the pairwise comparison of immunofluorescence and Western blot analyses. The following mouse lines were used: *Pten*-*loxP* mice^54^ and *proopiomelanocortin* (*Pomc*) promoter-driven *Cre* transgenic mice (*Pomc-Cre*^*Tg*/+^)^55^. *Pomc-Cre* transgenic (Tg) mice were purchased from Jackson Laboratory (Bar Harbor, ME, USA). Details for mating scheme are summarized in Supplementary Fig. S1. Littermates without the *Pomc-Cre* allele (*Pomc-Cre*^+/+^*;Pten*^*flox*/*flox*^) were used as controls. The primers that were used for genotyping are provided in Supplementary Methods.

### Video-monitoring EEG

Deeply anesthetized 8-week-old *Pten*-cKO mice underwent the chronic implantation of EEG electrodes, as previously described^56^. Electroencephalograms (EEG), which were recorded by the surface and depth electrodes, were analyzed (PowerLab/8sp, AD Instruments, Bella Vista) and stored using a commercial personal computer-based system, NB75H (Fujitsu, Tokyo). The animals’ behavior was monitored with a digital video camera (DCR-TRV9NTSC, Sony, Tokyo) for 8 hours per day. Epileptiform discharges were determined as clear high amplitude spikes and rhythmic waves or polyspikes^56^. Details in surgical procedures for electrode placement are described in Supplementary Methods.

### Quantitative real time PCR

Isolated brains were immediately cut on ice to separate the cortex, hippocampus, and hypothalamus. The dissected tissues were snap-frozen in liquid nitrogen and stored until use at −80 °C. Total RNA was extracted with an RNeasy Mimi Kit (Qiagen) for isolated frozen tissues. Complementary DNA was synthesized using a High-Capacity RNA to cDNA Kit (Life Technologies) according to the manufacturer’s protocol. A quantitative real time PCR (qRT-PCR) was performed using Fast SYBR Green Master Mix (Life Technologies) with custom primers (Supplementary Table S1) and a TaqMan Gene Expression Assay (Life Technologies) to identify *Pomc* expression, using the StepOnePlus system (Life Technologies). Mouse *Actb* was used as an internal control. Relative gene expression was calculated by the ddCt method.

### Immunofluorescence study

Immunofluorescence studies were performed as previously described^57^. Briefly, animals were deeply anesthetized and perfused with 4% paraformaldehyde (PFA) in phosphate buffered saline (PBS). The brain was removed and immersed in 4% PFA overnight. The fixed brains were cryo-protected with 20 and 30% sucrose-containing PBS at 4 °C for 24 hours each, and frozen in O.C.T. compound (Sakura Finetek, Tokyo). Fixed samples were serially cut at 40 μm of thickness. Sections were blocked with Block Ace (Morinaga, Japan) and incubated overnight at 4 °C with primary antibodies (See Supplementary Table S2 for the list of primary antibodies). Alexa-488 and 555 (Life Technologies, Carlsbad, CA) were used for the secondary antibodies. DAPI was used for nuclear staining. The signal intensity of an immuno-labeled protein in the region of interest (ROI) was measured for a quantitative analysis using the NIS-elements AR software program (Nikon Corporation, Tokyo, Japan) and the levels in the control and *Pten*-cKO mice were compared. Morphological analysis was performed as described in Supplementary Methods.

### BrdU labeling

The standard BrdU labeling methods that were used have been described previously^58^. Briefly, P14 and P28 mice were intraperitoneally injected with 50 μg/g of BrdU (Sigma-Aldrich Japan, Tokyo, Japan) twice at an interval of 8 h, and subjected to a histological analysis. Anti-BrdU monoclonal antibody was purchased from Roche Diagnostics Japan (Tokyo, Japan). A confocal laser scanning microscope system (A1 series, Nikon) was used to capture the microscopic images.

### Western blotting

Isolated brain tissues were homogenized in ice-cold, 0.32 M sucrose buffer containing Protease Inhibitor Cocktail (Nacalai Tesque, Kyoto) and Phos STOP Phosphatase Inhibitor Cocktail (Roche). Standard techniques for western blotting were used, and detailed procedures are described in Supplementary Methods.

### Drug administration

Rapamycin was injected systemically using a previously reported dosage regimen^59^. Rapamycin (LC Laboratories) was initially dissolved in 100% ethanol to a 20 mg/ml stock solution. Before injection, the stock solution was diluted in 5% Tween 80 and 5% polyethyleneglycol 400 to final concentrations of 5 mg/ml rapamycin. Rapamycin (10 mg/kg/day) or vehicle alone was intraperitoneally administered 5 days per week to mice of 6 to 9 weeks of age.

### Statistical analysis

All of the statistical analyses were performed using the JMP software program (SAS Institute, Cary, NC). The collected data are presented as means ± SD unless otherwise stated. The Wilcoxon rank sum test was used as a non-parametric method. P values of < 0.05 were considered to indicate statistical significance.

## Additional Information

**How to cite this article**: Matsushita, Y. *et al.* Hyperactive mTOR signals in the proopiomelanocortin-expressing hippocampal neurons cause age-dependent epilepsy and premature death in mice. *Sci. Rep.* **6**, 22991; doi: 10.1038/srep22991 (2016).

## Supplementary Material

Supplementary Information

Supplementary Video S1

Supplementary Video S2
