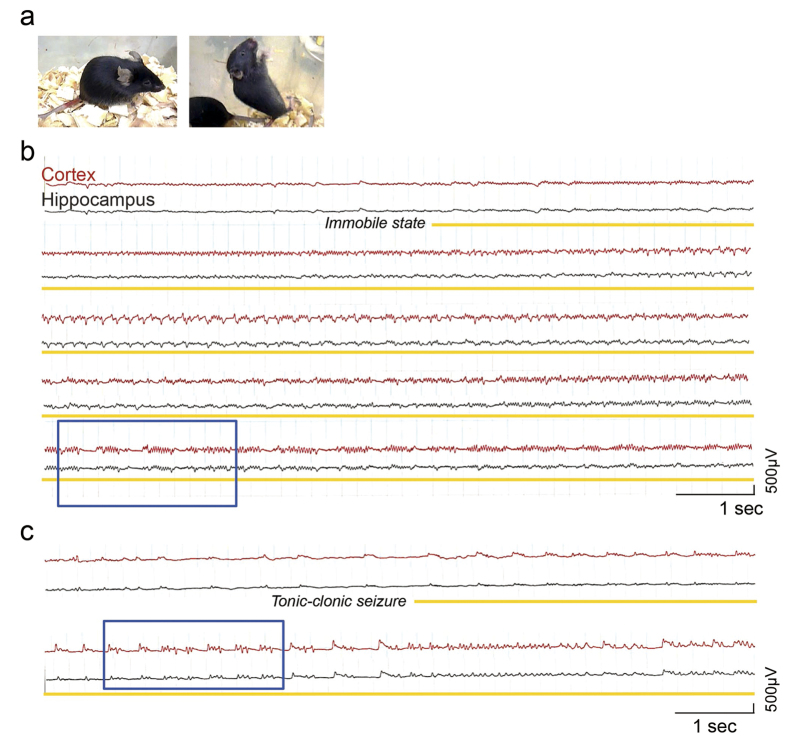# Corrigendum: Hyperactive mTOR signals in the proopiomelanocortin-expressing hippocampal neurons cause age-dependent epilepsy and premature death in mice

**DOI:** 10.1038/srep27164

**Published:** 2016-06-10

**Authors:** Yuki Matsushita, Yasunari Sakai, Mitsunori Shimmura, Hiroshi Shigeto, Miki Nishio, Satoshi Akamine, Masafumi Sanefuji, Yoshito Ishizaki, Hiroyuki Torisu, Yusaku Nakabeppu, Akira Suzuki, Hidetoshi Takada, Toshiro Hara

Scientific Reports 6: Article number: 2299110.1038/srep22991; published online: 03102016; updated: 06102016.

In Figure 2b and 2c, the electroencephalogram scale ‘500 μV’ is incorrectly given as ‘500 mV’. The correct Figure 2 appears below as Figure 1.

## Figures and Tables

**Figure 1:**