# Supplementary material for: Hyperactive mTOR signals in the proopiomelanocortin-expressing hippocampal neurons cause age-dependent epilepsy and premature death in mice
Source: Sci Rep. 2016 Mar 10;6:22991. doi: 10.1038/srep22991 (PMC4785342; doi:10.1038/srep22991)
Supplement: Supplementary Information [file srep22991-s1.pdf]

Supplementary Information for:

Hyperactive mTOR signals in the proopiomelanocortin-expressing hippocampal neurons cause age-dependent epilepsy and premature death in mice

Yuki Matsushita, Yasunari Sakai, Mitsunori Shimmura, Hiroshi Shigeto, Miki Nishio, Satoshi Akamine, Masafumi Sanefuji, Yoshito Ishizaki, Hiroyuki Torisu, Yusaku Nakabeppu, Akira Suzuki, Hidetoshi Takada, Toshiro Hara

This file contains:
Supplementary Figures S1-S7 and their legends
Supplementary Tables S1, S2
Supplementary Methods
Captions for Supplementary Videos S1, S2

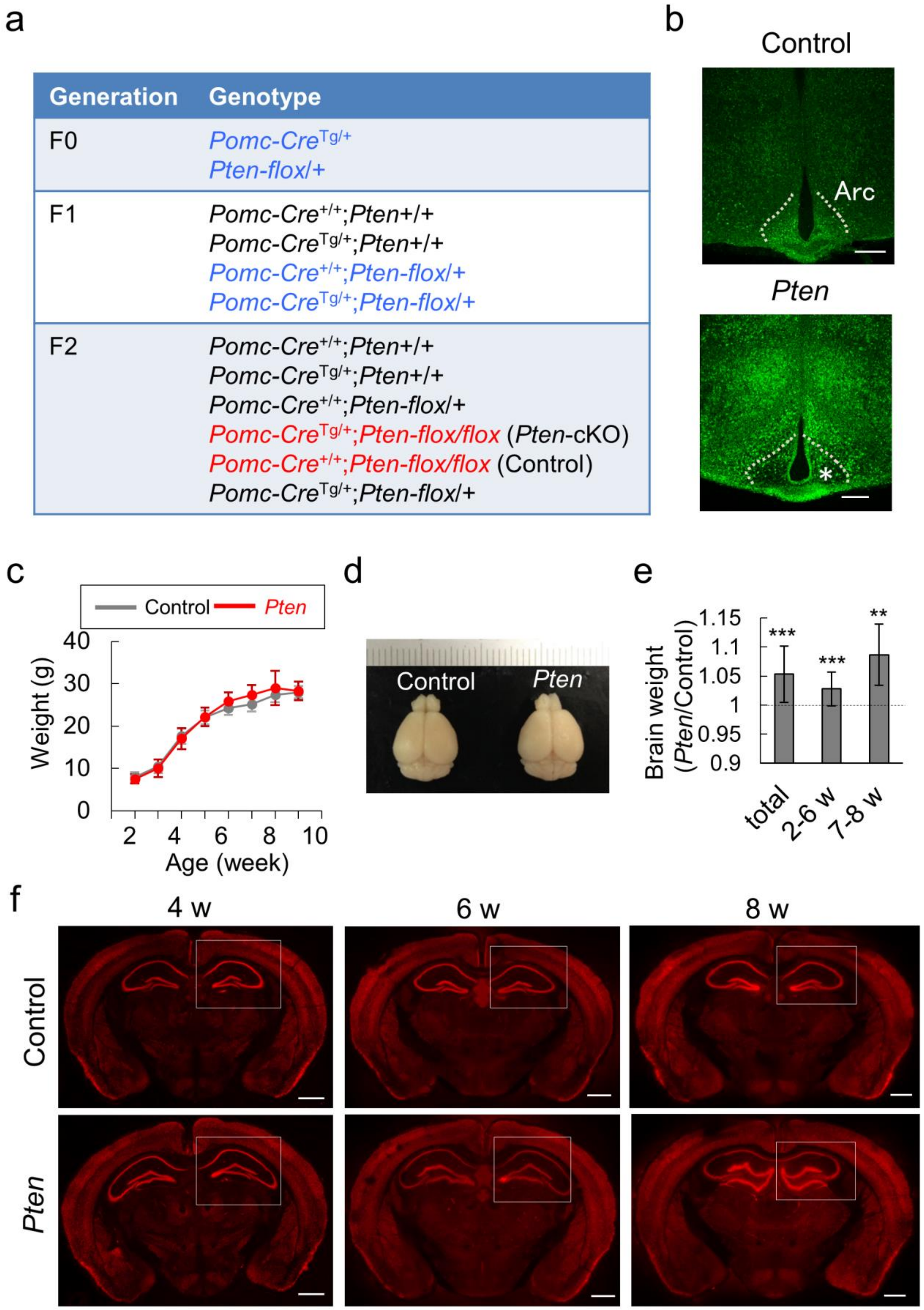
a
Generation
Genotype
F0
Pomc-Cre$^{Tg/+}$
Pten-flox/+
F1
Pomc-Cre$^{+/+}$;Pten+/+
Pomc-Cre$^{Tg/+}$;Pten+/+
Pomc-Cre$^{+/+}$;Pten-flox/+
Pomc-Cre$^{Tg/+}$;Pten-flox/+
F2
Pomc-Cre$^{+/+}$;Pten+/+
Pomc-Cre$^{Tg/+}$;Pten+/+
Pomc-Cre$^{+/+}$;Pten-flox/+
Pomc-Cre$^{Tg/+}$;Pten-flox/flox (Pten-cKO)
Pomc-Cre$^{+/+}$;Pten-flox/flox (Control)
Pomc-Cre$^{Tg/+}$;Pten-flox/+
b
Control
Arc
Pten
*
c
Control
Pten
Weight (g)
40
30
20
10
0
2
4
6
8
10
Age (week)
d
Control
Pten
e
Brain weight
(Pten/Control)
1.15
1.1
1.05
1
0.95
0.9
***
***
**
total
2-6 w
7-8 w
f
4 w
6 w
8 w
Control
Pten

Supplementary Figure S1. Overgrowth of the *Pten*-cKO brain

(a) The mating scheme. By mating the *Pomc-Cre*$^{Tg/+}$ and *Pten-flox/+* lines (F0), heterozygous (*Pomc-Cre*$^{Tg/+}$;*Pten+/+ and Pomc-Cre*$^{+/+}$;*Pten-flox/+*) and double-heterozygous mice (*Pomc-Cre*$^{Tg/+}$;*Pten-flox/+*) were obtained (F1). The next round of mating yielded *Pten*-cKO (*Pomc-Cre*$^{Tg/+}$;*Pten-flox/flox*) and control mice (*Pomc-Cre*$^{+/+}$;*Pten-flox/flox*) (F2). The mated pairs (blue) and the mice used in this study (red, control and *Pten*-cKO mice) are highlighted with colored fonts.

(b) Deficient Pten expression in the hypothalamic arcuate nuclei of *Pten*-cKO mice. Coronary sections from 4-week-old control and *Pten*-cKO mice (*Pten*), were dissected. Asterisks indicate the ablated Pten signals (green) in the arcuate nuclei (Arc) of a *Pten*-cKO mouse. Dashed lines indicate the boundary of the Arc. Scale bar, 200 μm.

(c) The growth curve for control (n = 31) and *Pten*-cKO male mice (n = 28).

(d) Whole brains dissected from control and *Pten*-cKO mice at 8 weeks of age.

(e) The overgrowth of the *Pten*-cKO brains. The relative ratios of the brain weight for the *Pten*-cKO mice (n = 18) to that of control mice (n= 15) are shown in the bar plot. Brains from *Pten*-cKO mice weighed significantly more than the brains of littermate controls at two different time points (Wilcoxon rank sum test; P values of <0.0001 in total, 0.0002 at 2-6 weeks, and 0.0028 at 7-8 weeks of age). **P <0.01, ***P <0.001. The plotted values show the means ± SD.

(f) An overview of the NeuN signals (red) in the whole coronary sections from the control (upper) and *Pten*-cKO (lower) mice at different ages. Note that the DG became prominently hypertrophic at 4-6 weeks of age. Scale bar, 1 mm.

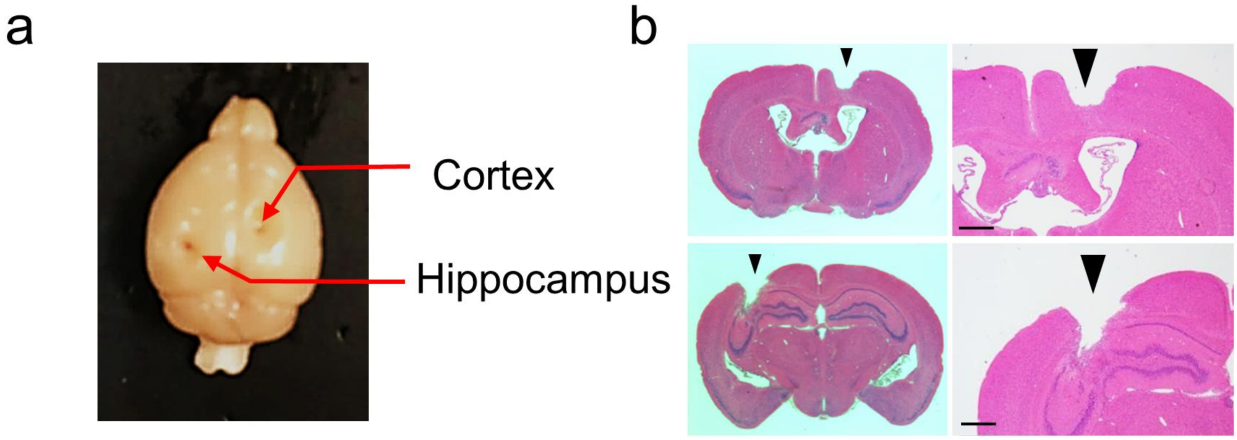

Supplementary Figure S2. The post-operational dissection of the *Pten*-cKO mouse

brain

(a) The whole-brain appearance of a *Pten*-cKO mouse after EEG recording at 10 weeks of age. Annotations indicate the positions of the cortical and hippocampal electrodes.

(b) The HE staining of a coronal section shows that the surface and deep electrode were both correctly inserted into the cerebral cortex (upper) and hippocampus (lower). The insertion points of the electrodes (arrowheads) are shown with higher magnification in the two images on the far right. Scale bar, 500 μm.

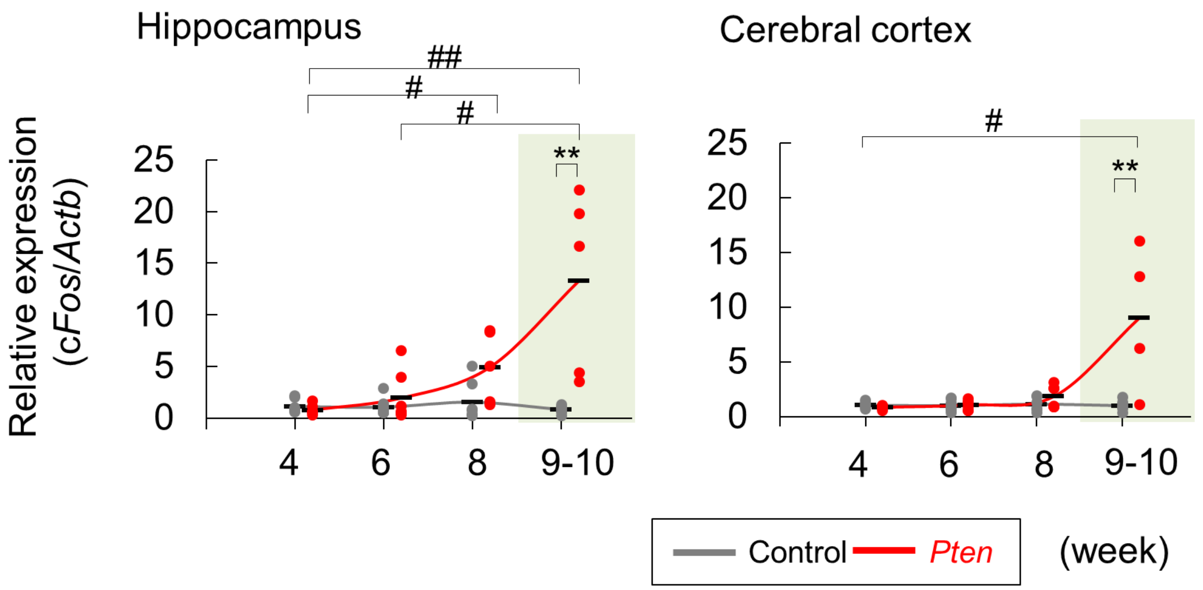

Supplementary Figure S3. The time course of *cFos* expression in the hippocampus and cerebral cortex

The increase in *cFos* mRNA expression in the hippocampus (left) of the *Pten*-cKO mice preceded that in the cerebral cortex (right). The control mice did not show such a similar increase in this period (4-10 weeks). The plots present the data from 3 or more pairs of littermates at each time point. The horizontal bars denote the average of the plots. Asterisks (*) indicate a statistically significant difference between different genotypes. Hashes (#) indicate a statistically significant difference between different time points. P < 0.05 (* and #), P < 0.01 (** and ##). *cFos* expression was normalized with the expression of *Actb*.

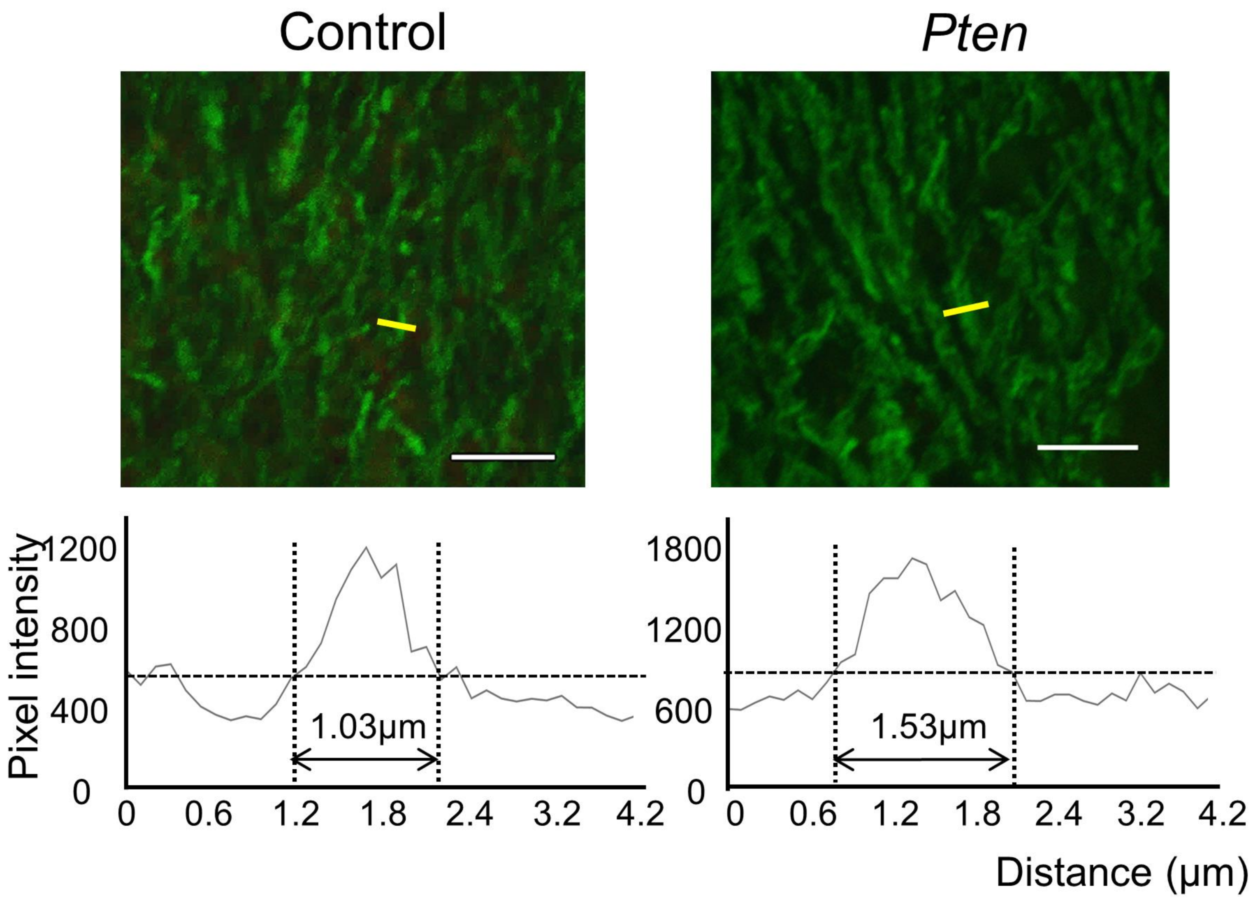

Supplementary Figure S4. The quantitative analysis of dendrite width

The upper panels show the fluorescence signal of Map2 (green) in the molecular layer of the DG. Four-week-old control and *Pten*-cKO mice were used for this assay. Optical cross-sections were set at right angles to the long axis of the dendritic shaft for the quantitative analysis of the dendritic width (yellow lines). The lower panels depict the contour plots for the quantitated Map2 signal. The width of each dendrite was measured using the standard full width at half maximum (FWHM) method, as shown in the lower panels. Scale bar (white line), 10 μm.

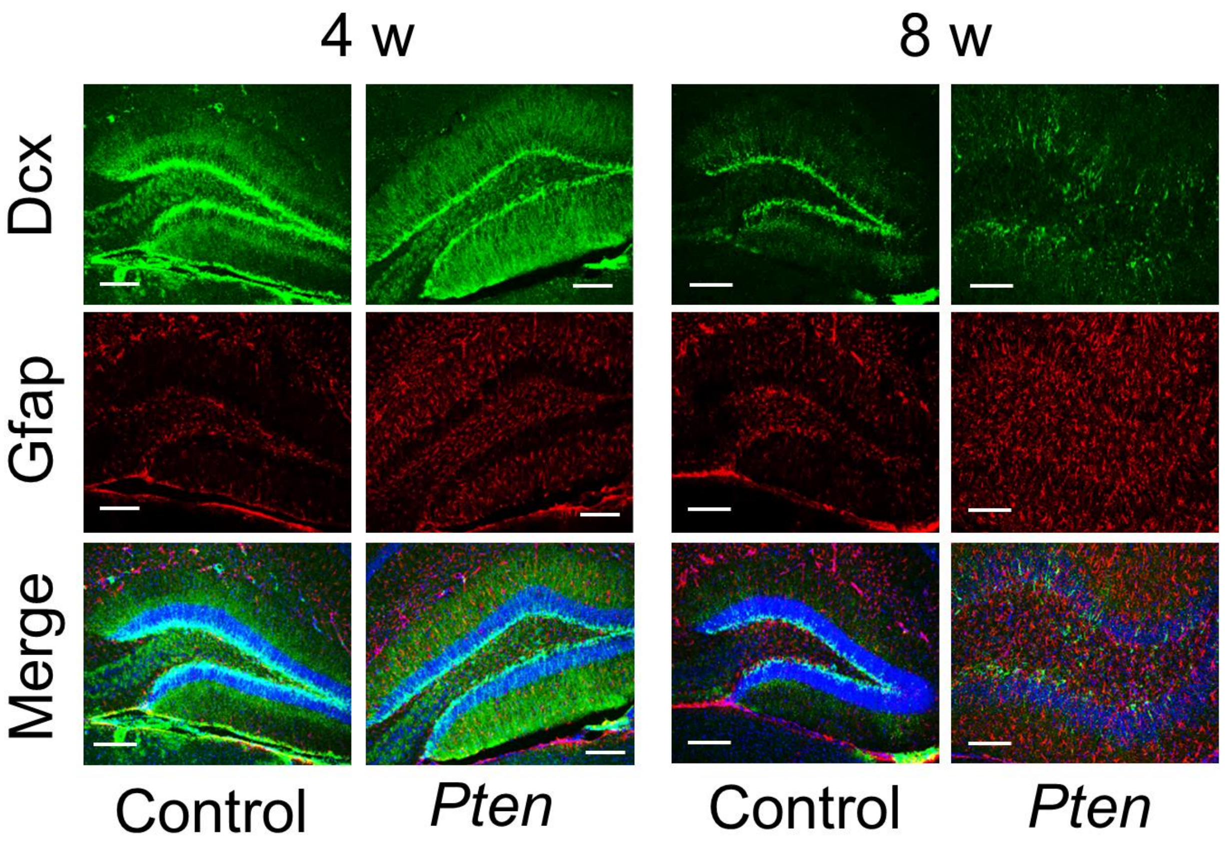

Supplementary Figure S5. Impaired neuronal differentiation in the hippocampal DG of *Pten*-cKO mice

The panels show the Dcx (green) and Gfap (red) signals in the DG of control and *Pten*-cKO mice at 4 and 8 weeks of age. Note that the Dcx signals peak at the subgranular layer in the control mice, whereas the Dcx signals are sparse in the DG of *Pten*-cKO mice at 8 weeks of age. The progressive increases in the Gfap signals during this timeframe are also presented. Scale bar, 200 µm.

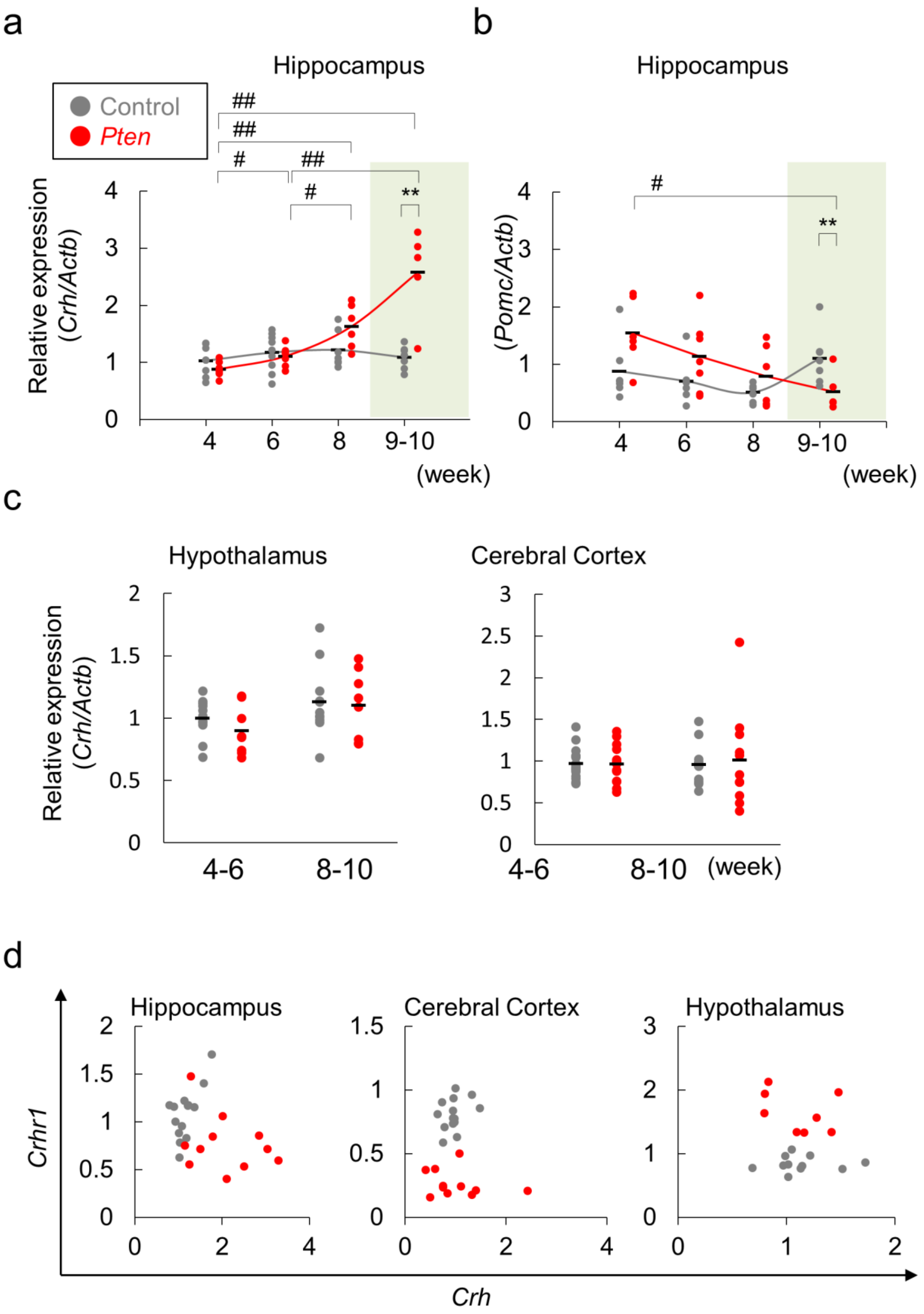
a
b
Hippocampus
Hippocampus
Control
Pten
##
##
#
##
#
**
**
Relative expression
(Crh/Actb)
(Pomc/Actb)
4
3
2
1
0
4
6
8
9-10
(week)
c
Hypothalamus
Cerebral Cortex
Relative expression
(Crh/Actb)
2
1.5
1
0.5
0
3
2.5
4-6
8-10
(week)
d
Hippocampus
Cerebral Cortex
Hypothalamus
Crhr1
Crh

Supplementary Figure S6. Aberrant *Crh, Pomc* and *Crhr1* expression in *Pten*-cKO mice

(a, b) The time course of *Crh* (a) and *Pomc* (b) mRNA expression in the hippocampus. Asterisks (*) indicate a statistically significant difference between different genotypes. Hashes (#) indicate a statistically significant difference between different ages. # P < 0.05; ** and ## P < 0.01.

(c) Unaltered *Crh* expressions in the hypothalamus and cerebral cortex of control and *Pten*-cKO mice before and after the onset of seizures.

(d) The co-expression profiles for *Crh* and *Crhr1* mRNAs in the hippocampus, cerebral cortex and hypothalamus of control and *Pten*-cKO mice.

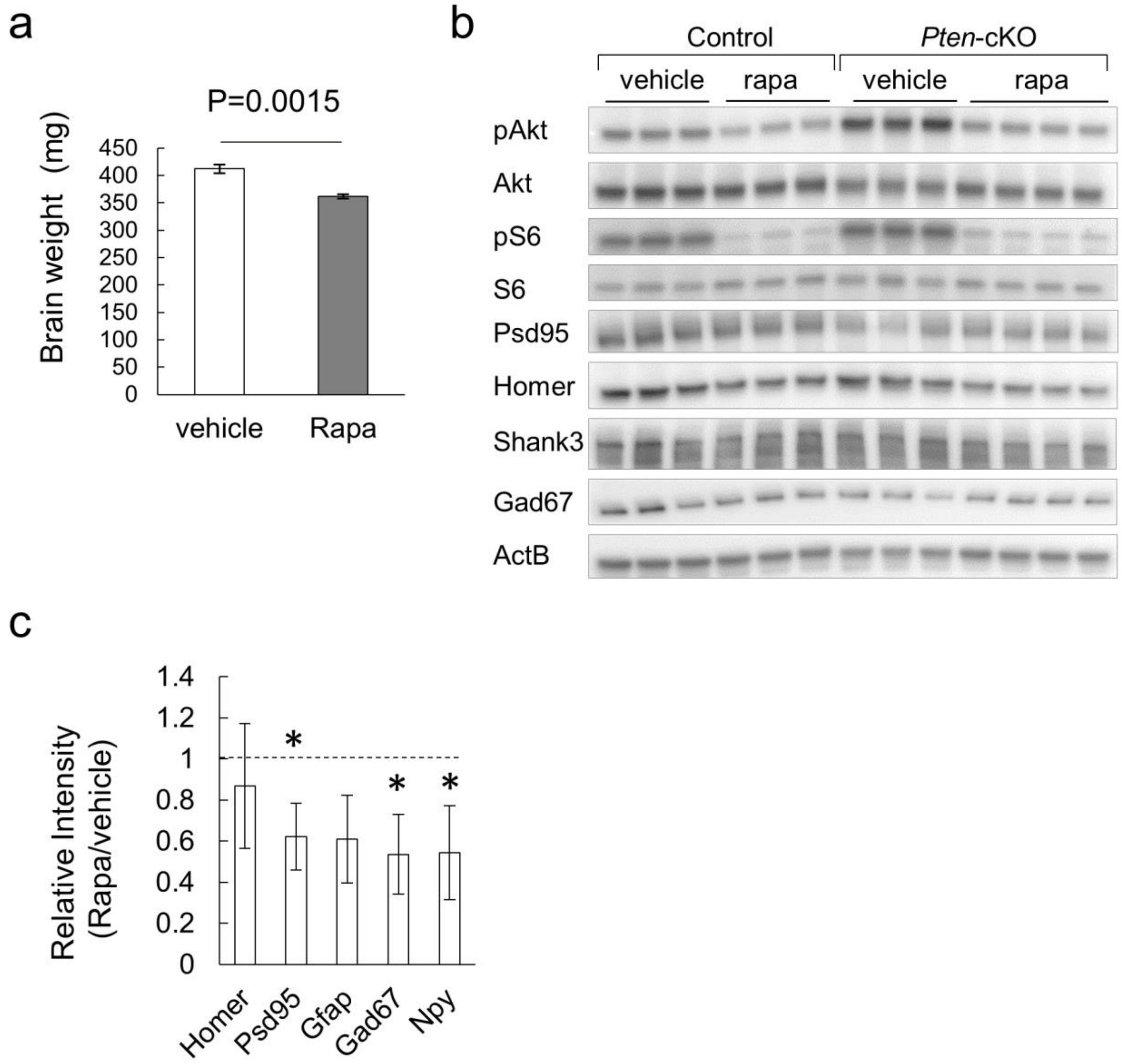

Supplementary Figure S7. Rapamycin reverses the phenotypes of *Pten*-cKO mice

(a) The total weight of the brain from vehicle or rapamycin-treated *Pten*-cKO mice. Whole brains were dissected at 9 weeks of age after vehicle (n = 3) or rapamycin (n = 3) treatment.

(b) Western blotting for the hippocampal extracts from vehicle- or rapamycin-treated *Pten*-cKO mice. The protein signals on these blots were used for a quantitative analysis. The measured signal intensity for each protein was normalized with that of Actb. The mean values obtained from vehicle-treated control mice were used as reference values (value: 1 for each protein). The quantitated data were plotted (Fig. 5b).

(c) The relative fluorescence signals of the marker proteins. Three pairs of *Pten*-cKO mice with or without rapamycin treatment were subjected to an immunofluorescence analysis. The fluorescence signals of each protein in the regions of interest (squared regions in Fig. 5d) were measured and plotted as the mean ± SD. * $P < 0.05$.

Supplementary Table S1. The oligonucleotide primers used for the quantitative PCR analyses

Gene	Forward (5' to 3')	Reverse (5' to 3')
Pten	CGGAACTTGCAATCCTCAGT	AATGGCTGAGGGAACTCAAA
Gfap	AAGCCAAGCACGAAGCTAAC	CATTTGCCGCTCTAGGGACTC
Psd95	GCCTAAAGGACTTGGCTTCA	GGATCTTGTCTCCGATCTGC
Shank3	TGCAGCAGCTGAATAAAGACA	TGAGATGGTGCTCAGCTCAC
Shank1	CAAAGACTGGGTTGAAGAAG	GGTGTCTCTCCTGAATCTGA
Homer1	CAGTTTAGATGGCTCAAAGG	AAGATGATGCTCAGAGGAGA
Grm5	AGCAAGTGATCAGAAAGACTCG	GTCACAGACTGCAGCAGAGC
Cntnap2	CTTGGCACCTAGATCACTTG	CCTCCAATGATAGCTGAGTT
Nlgn3	CATCCTGTGTCAGTCTCCTT	TCACTGGTTGGTAGTTCACA
Pvalb	CGCTGAGGACATCAAGAAGG	CCGGGTTCTTTTTCTTCAGG
Gad67	CAAACTCAGCGGCATAGAAA	GAAGAGGTAGCCTGCACACA
Gphn	AGGATCTCAGGAATGCTTTC	CTTCAAGTTCATCATGCACC
Crh	GCAGTTAGCTCAGCAAGCTCAC	CAAATGATATCGGAGCTGCG
Crhr1	AAGTGGATGTTCGTCTGCAT	CCAAACCAGCACTTTTCATT
Mc4r	TCTCTATGTCCACATGTTCCTG	GGGGCCCAGCAGACAACAAAG
cFos	GAGGAAGAGAAACGGAGAAT	GTCTCCGCTTGGAGTGTAT
Actb	TTGGGTATGGAATCCTGTGG	CTTCTGCATCCTGTCAGCAA

Supplementary Table S2. The primary antibodies used in the present study

Antigen	Host	Supplier	Cat.#	IF	WB
Pten	Rb	Cell Signaling Technology	#9188	1/100	1/1000
Akt	Rb	Cell Signaling Technology	#4691	1/1000	1/1000
pAkt(Ser473)	Rb	Cell Signaling Technology	#4060	1/100	1/2000
S6	Rb	Cell Signaling Technology	#2217	1/1000	1/1000
pS6(Ser240/244)	Rb	Cell Signaling Technology	#5364	1/1000	1/1000
NeuN	Ms	Merck Millipore	MAB377	1/3000	-
cFos	Rb	Santa Cruz Biotechnology	sc-52	1/1000	-
Npy	Rb	Santa Cruz Biotechnology	sc-133080	1/100	-
Homer	Rb	Santa Cruz Biotechnology	sc-15321	1/500	1/200
Psd95	Ms	Abcam	ab2723	1/1000	1/500
Gfap	Ms	Sigma	G3893	1/5000	1/5000
Shank3	Rb	Santa Cruz Biotechnology	sc-30193	-	1/200
Gad67	Ms	Millipore	MAB5406	1/15000	1/5000
Map2	Rb	Abcam	ab32454	1/100	-
BrdU	Ms	Roche Diagnostic Japan	BMC9318	1/1000	-
Dcx	Goat	Santa Cruz Biotechnology	sc-8066	1/100	-
Znt3	Rb	Synaptic Systems	197 002	1/500	-

IF, immunofluorescence; WB, western blot; -, not used

Supplementary Methods

Genotyping

Genotyping PCR using the genomic DNA was performed as described (Chao et al., 2010). Following primers to amplify transgene (Cre) and internal positive control gene (PC) were used: (Cre-for) 5'-CCATCTGCCACCAGCCAG-3' and (Cre-rev) 5'-TCGCCATCTTCCAGCAGG-3'; (PC-for) 5'-ACTGGGATCTTCGAACTCTTTGGAC-3' and (PC-rev) 5'-GATGTTGGGGCACTGCTCATTCACC-3'. *Pten-flox* alleles were detected with the following primers (Flox-for) 5'-GTGAAAGTGCCCCAACATAAGG-3' and (Flox-rev) 5'-CTCCCACCAATGAACAAACAGTC-3'.

Surgical operation for the electrode placement

A mouse was fixed on a stereotactic frame and the head skin was cut to expose the cranium. A stainless steel screw electrode was placed at subdural point of the right parietal lobe (1 mm posterior to the Bregma and 1 mm lateral from the midline). A depth-recording electrode, which was made of twisted nichrome wire (diameter 0.02 mm), was inserted into the CA3 regions of the left hippocampus (3 mm posterior from the Bregma, 3 mm lateral to the midline, and 2 mm deep from the cortical surface). A reference screw electrode was placed on the parietal bone. All electrodes were connected to a 2-channel amplifier (MEG-6168, Nihon Kohden, Tokyo).

Western blotting

Twenty-five µg of total protein was applied for SDS-PAGE using 4-15% Mini-PROTEAN TGX Gels (BioRad). Separated proteins were transferred to PVDF membranes (Trans-Blot Turbo Transfer Pack, BioRad). The blotted membranes were blocked with 5% milk and incubated overnight at 4˚C with primary antibodies (Supplementary Table S2). Secondary antibodies conjugated to horseradish peroxidase (211-032-171 or 115-035-174, Jackson ImmunoResearch, West Grove, PA) were used to detect chemiluminescence (Clarity ECL substrate, BioRad, and ImmunoStar LD, Wako). All measurements were performed using the FluorChem FC2 System software program (ProteinSimple, San Jose, CA, USA). Beta-Actin (Actb) was used as an internal control.

Morphological analysis

Neurons in the granule cell layer of the dentate gyrus were analyzed with a confocal laser microscopic system. For each targeted region of interest, a total of 20 XY-plane images were captured at 0.13 µm per step for the Z-axis (2.6 µm of thickness) with a 100x objective lens (Nikon, CFI Plan Apo VC 100xH). Stacked images were projected onto one XY-plane,

and the resulting image was used for the morphological analyses. The dendrite width was measured with the full width at half maximum (FWHM) method, as previously described [1]. Briefly, 15-20 regions of interest (ROIs) were set on an XY plane, and the Map2 signal intensity was converted to a contour-tracing plot on an optical cross-section of a target dendrite. The cross-section line for a dendritic shaft was drawn with a right angle to the longitudinal axis of the target dendrite (Supplementary Fig. S4). The size of the soma was determined by measuring the total surface area of nuclei with NeuN-positive signals in the granule cell layer using the NIS-elements AR software program (Nikon corporation, Tokyo, Japan). Distances between two nuclei were also measured for each ROI and the results were consistent with an independent method based on the size of the NeuN-positive nuclei. Data from 12 confocal images were collected from at least 3 pairs of *Pten*-cKO and control littermates.

Supplementary Reference

1. Nagerl UV, Willig KI, Hein B, Hell SW, Bonhoeffer T. Live-cell imaging of dendritic spines by STED microscopy. *Proc Natl Acad Sci U S A* **105,** 18982-18987 (2008).

Caption for Supplementary Videos.

The two types of seizure pattern. Two movies are provided to show the unique seizure patterns of a female *Pten*-cKO mouse at nine weeks of age. Digital video images were processed using the Super Media Converter software program (Wondershare Software, Tokyo). The EEG recordings for Supplementary Video S1 and S2 are presented in Fig. 2b and Fig. 2c, respectively.

Supplementary Video S1. The unresponsive, immobile seizure pattern: This movie (0'40'') starts with the normal behavior of a *Pten*-cKO mouse (0'00''-0'04''). The mouse stops moving with intermittent sniffing.

Supplementary Video S2. A generalized tonic-clonic seizure: This movie (0'38'') shows a generalized tonic-clonic seizure (0'13''-0'32'') which includes head nodding, tonic posture, ramping and falling backwards with forelimb clonus. The seizure ends with the mouse standing up again, breathing heavily and intermittent forelimb clonus.
